# Overexpression of JARID1B promotes differentiation via SHIP1/AKT signaling in human hypopharyngeal squamous cell carcinoma

**DOI:** 10.1038/cddis.2016.262

**Published:** 2016-09-01

**Authors:** Jisheng Zhang, Xiaofei An, Yafei Han, Rui Ma, Kun Yang, Lu Zhang, Jingwei Chi, Wei Li, David Llobet-Navas, Yan Xu, Yan Jiang

**Affiliations:** 1 Key Laboratory, Department of Otolaryngology-Head and Neck Surgery, Affiliated Hospital of Qingdao University, Qingdao, Shandong 266003, China; 2 Department of Endocrinology, Jiangsu Province Hospital of Chinese Medicine, 155 Han Zhong Road, Nanjing 210029, China; 3 Department of Nephrology, Affiliated Hospital of Qingdao University, Qingdao 266 003, China; 4 Institute of Genetic Medicine-Newcastle University, Newcastle upon Tyne NE1 3BZ, UK

## Abstract

Histone H3 (H3K4) demethylase JARID1B is aberrantly upregulated in many types of tumor and has been proposed to function as oncogene. Here we show that JARID1B is elevated in moderate and high-differentiated human hypopharyngeal squamous cell carcinoma (HPSCC) compared with low-differentiated HPSCC. Overexpression of JARID1B in FaDu cells increased epithelial differentiation marker K10 expression and inhibited cell proliferation. JARID1B and K10 mRNA expression is high correlated in HPSCC patients. Mechanistically, we found JARID1B directly bound to PI3K/AKT signaling inhibitor SHIP1 gene promoter and decreased SHIP1 gene expression. Activation of downstream AKT resulted in increased *β*-catenin signaling, by which promoted target genes Fra-1 and Jun, together with other AP-1 transcription factors, leading to K10 expression. Forced expression of SHIP1 rescued JARID1B-induced phenotypes on FaDu cell differentiation and proliferation. Taken together, our findings provide first evidence that elevated expression of JARID1B has a critical role in promoting HPSCC differentiation and inhibiting proliferation, suggesting JARID1B may function as a tumor suppressor in squamous cell cancers and implying a novel important therapeutic strategy of HPSCC.

Hypopharyngeal squamous cell carcinomas (HPSCC) have the worst survival though it only represents 3–4% of all head and neck cancers.^1^ Studies demonstrated that early-stage HPSCC has a 71% 5-year survival rate.^2^ However, once up to advanced stage 5-year survival rate could be as low as 16% and as high as 47%.^3, 4^ HPSCC usually does not give rise to symptoms until late in the course of the disease. To date, treatment of HPSCC is still a big challenge and less attention has been received, in part because of its low incidence and clinical difficulty. HPSCC can be treated by surgery on the early stage, whereas a large part of patients was found to have late-stage cancer at diagnosis.^5^ In recent years, instead of surgical excision non-surgical treatments have been used to largely preserve organ function. It has been reported that there is no significant survival differences between the organ preservation and surgery arms,^6^ suggesting the importance of non-surgical treatment in this type of cancer. Therefore, it is important and necessary to develop alternative treatment strategies. Recent decades the growing understanding of molecular mechanisms involved in the cancer cell proliferation, differentiation and cell death has led to the development of multiple targeted therapies with significant clinical benefits. However, little is known about the mechanisms involved in progression of HPSCC.

There is emerging evidence for a causal role of aberrant expression of Jarid1b in human cancer. Jarid1b belongs to Jarid1 subfamily, which comprises four members, JARID1A (KDM5A/RBP2), JARID1B (KDM5B/PLU1), JARID1C (KDM5C/SMCX) and JARID1D (KDM5D/SMCY). They share a JmjC (catalytic) domain and could demethylase H3K4me3 to produce histone H3 (H3K4)me2 and/then H3K4me1, resulting in gene transcription repression upon removal of H3K4me3. Jaird1b has been reported to be overexpressed in breast cancer,^7, 8, 9^ lung cancer, bladder cancer,^10, 11^ colorectal cancer,^12^ prostate cancer^13^ and malignant melanoma.^14^ Jarid1b is upregulated in these cancers and required for cancer cell proliferation and tumor growth. In breast cancer it has been reported that the depletion of Jarid1b led to inhibition of breast cancer cell proliferation and to reduce tumor growth in xenografts^15^ or syngeneic mouse mammary tumor model.^16^ Similar results were obtained in lung cancer, bladder cancer and colorectal tumors. Interestingly, Roesch *et al.*^17^ characterized a slowly cycling subpopulation of human melanoma cells that express high levels of Jarid1b, whereas this subpopulation cell can give rise to highly proliferative progeny with low Jarid1b expression. The dynamic regulation of Jarid1b expression suggests a new mechanism of Jarid1b-regulating stem cell-like properties in tumor maintenance. This JARID1B^high^ subpopulation is enriched upon a variety of drug treatments, which could be blocked by inhibition of mitochondrial respiration.^14^ The phenotypic consequences in various types of human cancer highlight importance of elevated Jarid1b expression. However, the mechanisms of Jarid1b controlling the described phenotypes are not fully understood yet. It seems that Jarid1b-regulating genes presents cell type-specific pattern.

Moreover, Jarid1b has an important role in cell differentiation during development. Jarid1b controls mesenchymal cell fate into myogenic and osteogenic lineages,^18^ macrophage^19^ and embryonic stem cell (ESC) differentiation.^20, 21^ In luminal breast tumors overexpressed Jarid1b functions as a luminal lineage-driving oncogene. High luminal JARID1B activity is correlated with poor outcome in patients with hormone receptor-positive breast tumors.^22^ However, the mechanisms of Jarid1b leading to the differentiation in tumors remain barely explored.

Here we investigated the function of Jarid1b elevation in HPSCC. We found that the high/moderate differentiated HPSCC had high Jarid1b expression compared with low-differentiated cancer. In FaDu cells overexpressed Jarid1b led to high expression of K10, which is an epithelial differentiation marker. The forced expression of JARID1B impaired cell proliferation, indicating that Jarid1b may function as a repressor of cancer progression. Mechanistically, our data showed that Jarid1b downregulated Ship1 and in turn activated PI3K-AKT pathway. Consequently, *β*-catenin was phrosphorlated by AKT and the *β*-catenin target genes Fra-1 and Jun, together with other AP-1 genes, induced K10 expression. Restoration of Ship1 could rescue the phenotypic consequences of Jarid1b overexpression. In this study, we mainly forced expression of Jarid1b to mimic the pathological process in HPSCC. Our findings reveal a novel function of Jarid1b in control of the tumor differentiation and may provide perspective to develop novel therapeutic interventions for HPSCC and other squamous cell carcinomas.

## Results

### Jarid1b is overexpressed in the moderate and high-differentiated HPSCC

**Figure 1:**
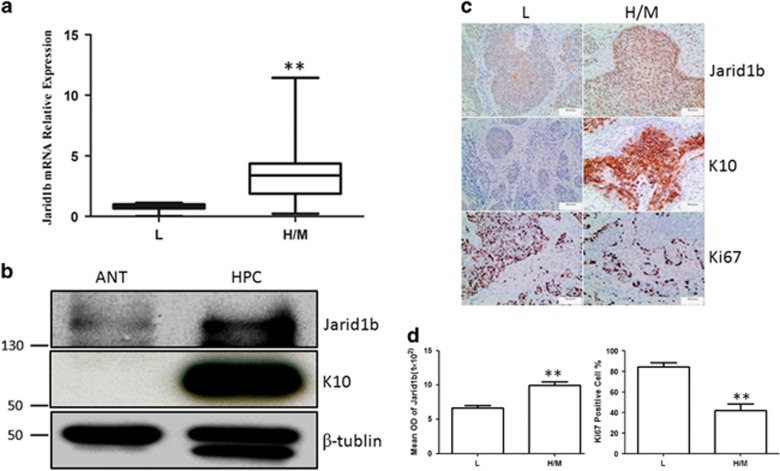
Jarid1b is overexpressed in the moderate and high-differentiated HPSCC. (**a**) Measurement of *Jarid1b* mRNA expression for the divided groups by quantitative RT-PCR. L: low-differentiated HPSCC (*n*=10), H/M: moderate and high-differentiated HPSCC (*n*=24). **P<0.01, Student's *t*-test. (**b**) JARID1B and K10 proteins were detected by immunoblotting in the H/M-differentiated HPSCC (HPC) and adjacent normal tissue (ANT). (**c**) Representative images of immunohistochemical staining for JARID1B, K10 and Ki67 in L and H/M-differentiated human HPSCC. K10 showed no obvious staining in cytoplasm in low-differentiated HPSCC. All scale bars are 100 *μ*m. (**d**) Quantifications were provided and showed high JARID1B expression and low Ki67-positive percentage in the H/M HPSCC. **P<0.01, Student's *t*-test

It has been reported that Jarid1b is upregulated in many types of cancer and promotes proliferation of cancer cells. We first used the RT-qPCR method to quantify the expression of *Jarid1b* in HPSCCs compared with the adjacent normal tissues. Unfortunately, there was no significant difference owing to the variation of *Jarid1b* expression level. Some recent researches have demonstrated that Jarid1b could be involved in differentiation during development. To gain the insights into the role of Jarid1b in the cancer differentiation, we divided all the samples into two groups according to the pathological differentiation grade diagnosis. We found that Jarid1b was high expressed in the moderate and high-differentiated HPSCC compared with the low-grade samples (Figure 1a). Consistently, the observation was confirmed by western blot that JARID1B was upregulated compared with the adjacent normal tissue in the moderate/high-differentiated HPSCC. In addition, K10, a specific epithelial differentiation marker, was also markedly elevated in the cancer (Figure 1b). To further examine role of Jarid1b regarding to differentiation and proliferation, we performed the IHC staining against Jarid1b, K10 and Ki67. Ki67 is an excellent marker to define the proliferation population and often correlated with the clinical course and outcomes of cancer. Compared with the low-grade cancer JARID1B was high expressed in the moderate and high-differentiated HPSCCs, which displayed strong K10 staining and low percentage of Ki67 (Figures 1c and d).

### Forced expression of Jarid1b leads to FaDu cell differentiation

**Figure 2:**
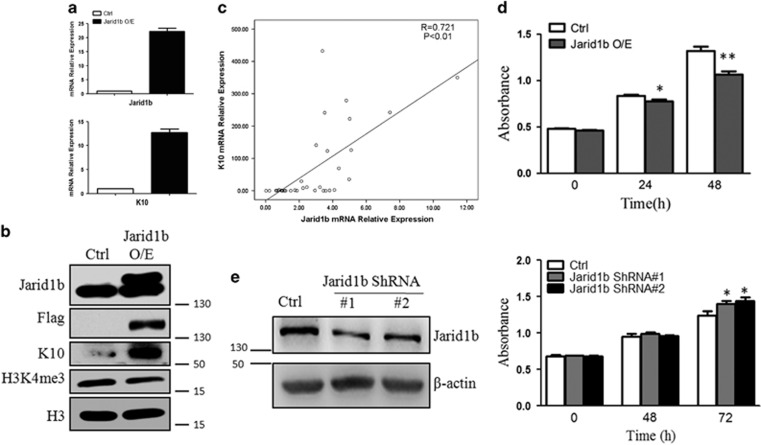
Forced expression of Jarid1b leads to FaDu cell differentiation. (**a**) K10 mRNA expression was assessed by RT-qPCR in stable Jarid1b-overexpressing (O/E) FaDu cells. (**b**) Protein lysates of FaDu cell-transfected Flag-Jarid1b or control were analyzed for JARID1B, Flag, K10, H3K4me3 and Histone 3(H3) by immunoblotting. (**c**) Correlation between *Jarid1b* gene expression (mRNA) and *K10* gene expression (mRNA) in all human HPSCCs (*n*=34) based on their gene expression analysis by RT-qPCR. (**d**) Cell proliferation was measured by CCK8 assay at indicated time points in Jarid1b or control-overexpressing cells. *P<0.05, **P<0.01, Student's *t*-test. (**e**) Cell proliferation was measured by CCK8 assay at indicated time points in Jarid1b or control knockdown cells. *P<0.05, Student's *t*-test

To assess the potential role of Jarid1b in the regulation of HPSCC differentiation, Flag-Jarid1b was overexpressed in FaDu cells, which came from a hypopharyngeal squamous cell carcinoma. As expected overexpression of Jarid1b increased K10 mRNA expression level in FaDu cells (Figure 2a). We tried to explore more epithelial keratin markers such as K13, but unfortunately we did not find any other reliable differentiation gene markers. Thus, in this study we only used K10 as a differentiation marker. Consistent with RT-PCR results, forced expression of Jarid1b led to elevation of K10 at protein level. In addition, H3K4me3 was downregulated upon Jarid1b expression (Figure 2b). To evaluate the significance of Jarid1b in control of K10 expression, we analyzed correlation between these two gene mRNA levels normalized to adjacent normal tissue in all the 34 HPSCC samples. High-positive correlation was obtained between mRNA levels of Jarid1b and K10 (*R*=0.721, *P*<0.01), suggesting that Jarid1b expression level is an important influential factor for K10 expression in HPSCC (Figure 2c). It has been well documented that Jarid1b depletion inhibited cancer cell proliferation. But how the overexpression of Jarid1b affects cell growth has been ignorable. We overexpressed Jarid1b or control in FaDu cells and exerted CCK8 assay to measure the cell proliferation. Unexpectedly, upregulation of Jarid1b inhibited FaDu cell proliferation compared with control (Figure 2d). The results are consistent with IHC staining, which showed that high-differentiated HPSCCs have high JARID1B and low Ki67 expression (Figures 1c and d). These observations suggested that Jarid1b may affect cancer cell growth via different mechanisms.

### Knockdown of Jarid1b represses K10 transcription

Next we knockdown Jarid1b with shRNA lentivirus to further validate the role of Jarid1b in control of FaDu cell differentiation. Jarid1b mRNA and protein expression has been markedly reduced after infection 72 h (Supplementary Figures S2A and 2e). Compared with control K10 mRNA expression decreased upon depletion of Jarid1b (Supplementary Figure S2B). However, K10 protein expression was undetectable even with long exposure, whereas much less-loaded HaCaT cells showed a very strong band (Supplementary Figure S2C). Our results are consistent with characteristic of poorly differentiated FaDu cell. Finally, CCK8 assay was performed to measure cell proliferation with Jarid1b depletion. Knockdown with Jarid1b shRNA lentivirus promoted cell proliferation compared with control (Figure 2e).

### *β*-catenin signaling is activated by overexpression of Jarid1b

**Figure 3:**
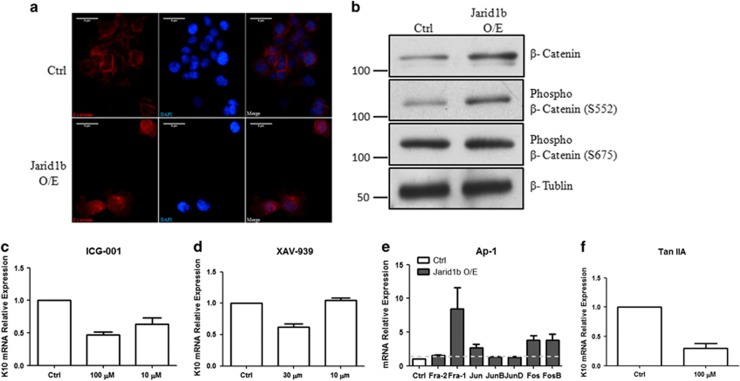
*β*-catenin signaling is activated by overexpression of Flag-Jarid1b. (**a**) Puromycin selected Jarid1b-overexpressing and control FaDu cells were stained with *β*-catenin antibody (red) and counterstained with DAPI, showing that Jarid1b O/E cells displayed nucleus staining pattern compared with control. (**b**) *β*-catenin and its two phosphorylated isoforms, S552 and S675, were detected by immunoblotting in stable Jarid1b O/E or control cells. (**c**) K10 mRNA expression was analyzed by RT-qPCR upon treatment of *β*-catenin inhibitors, ICG001 and (**d**) XAV-939 and (**f**) AP-1 inhibitor Tan IIA at indicated concentration in stable Jarid1b O/E cells. (**e**) Expression of indicated AP-1 genes was assessed by RT-qPCR in stable Jarid1b O/E and control cells

*β*-catenin (CTNNB1) signaling exerts crucial roles in the regulation of cell behaviors such as proliferation, survival and differentiation. We transiently overexpressed Flag-Jarid1b or control vector into FaDu cells and pure cell population was selected by puromycin. The cells were fixed and immnostained against *β*-catenin (red) antibody. In contrast to control cells, which showed cytoplasm and membrane staining pattern, the Jarid1b O/E cells displayed accumulation of *β*-catenin staining in the nucleus and cytoplasm (Figure 3a), indicating that overexpression of Jarid1b enhances the nuclear translocation of *β*-catenin and activates *β*-catenin signaling. By western blot higher levels of *β*-catenin were detected in Jarid1b-overexpressing cells. Interestingly, Jarid1b selectively promoted the phosphorylation of *β*-catenin at S552 but not S675, implying specific regulation manner of Jarid1b (Figure 3b). Treatment of *β*-catenin inhibitors, ICG001 and XAV-939, repressed K10 expression in stable Jarid1b-overexpressing cells (Figures 3c and d).

Two AP-1 transcription factors, Fra-1 and Jun, have been found to be downstream target genes of activated *β*-catenin.^23^ In addition, our previous results and others have demonstrated that Ap-1 is involved in regulating epithelial marker genes by direct promoter binding.^24, 25^ Indeed, many of AP-1 genes including Jun, Fos, FosB and Fra-1 showed high expression in the stable Jarid1b-overexpressing cells (Figure 3e). Moreover, treatment of Tanshinone IIA, which is thought to abrogate all AP-1 binding to DNA, decreased the expression of K10 (Figure 3f). These results suggest that *β*-catenin may have a critical role in regulating K10 expression.

### Overexpression of Jarid1b results in activation of AKT

**Figure 4:**
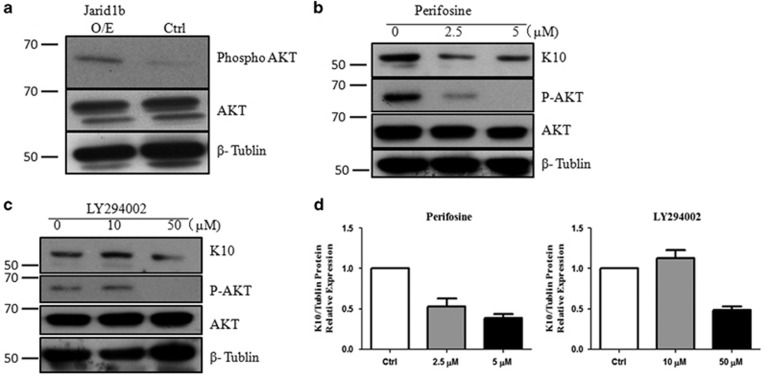
Overexpression of Jarid1b results in activation of AKT. (**a**) AKT and Phosphorylated AKT were detected by immunoblotting in stable Jarid1b O/E or control cells. (**b**) K10, AKT and Phosphorylated AKT were detected by immunoblotting upon the treatment of AKT inhibitor Perifosine and (**c**) PI3K inhibitor LY294002 at indicated concentration in stable Jarid1b O/E cells. (**d**) Quantifications were assessed from three independent experiments showing as fold change *versus* control

As Akt can phosphorylate *β*-catenin at S552 and promotes *β*-catenin nuclear localization,^26, 27, 28^ we tested whether the upregulation of Jarid1b influences Akt activation. As shown in Figure 4a, overexpression of Jarid1b led to increase of phosphorylated AKT though it did not affect the pan-AKT expression. In stable Jarid1b-overexpressing cells with adding specific inhibitors of AKT, Perifosine, we found that it significantly inhibited K10 production at either 5 or 2.5 *μ*m (Figures 4b and d), suggesting activated AKT is involved in K10 expression in response to Jarid1b overexpression. Next we tested the AKT upstream molecule PI3K by its inhibitor, LY294002, to further validate the promotive effect of Phosphor-AKT on K10 expression and underlying pathways. Consequently, we found that at 50 *μ*M concentration LY294002 could effectively decrease K10 protein level (Figures 4c and d). In the control cells we got similar results at transcription level with RT-qPCR though K10 protein expression was undetectable owing to poor differentiation of FaDu cells (Supplementary Figures S4A–D). These findings suggest that PI3K-AKT pathway is required for Jarid1b driving FaDu cell differentiation.

### Ship1 is downregulated upon Jarid1b overexpression

**Figure 5:**
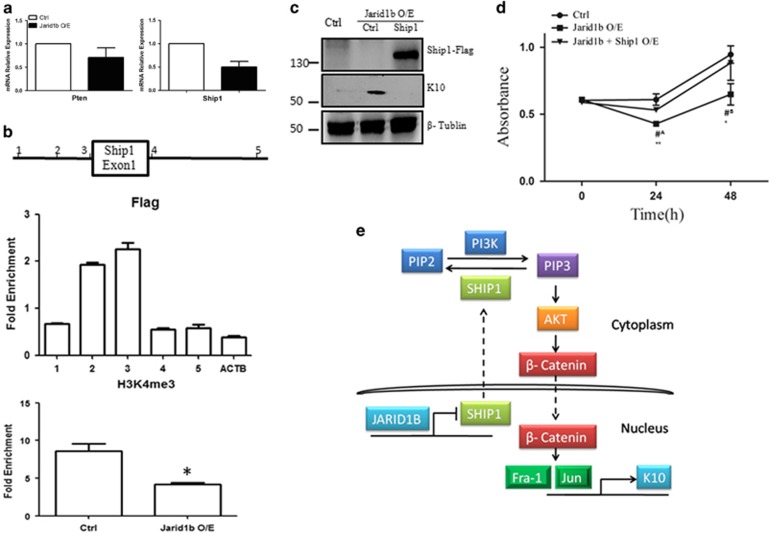
Jarid1b promotes FaDu cell differentiation through directly repression of *SHIP1* gene. (**a**) *Pten* and *Ship1* mRNA expression were analyzed by RT-qPCR in Jarid1b O/E and control cells. (**b**) ChIP studies on Jarid1b-overexpressing cells showed Jarid1b binding at the *SHIP1* promoter region. The scheme indicated the designed primer location at *SHIP1* gene. ChIP-qPCR signal was normalized to experimental control cells infected with control vector. (**c**) K10 and Flag were detected by immunoblotting in control and stable Jarid1b-overexpressing FaDu cells transfected with control vector or Ship1-Flag. (**d**) Cell proliferation was measured by CCK8 assay at indicated time points in Jarid1b and control or Ship1-Flag co-transfected cells. Jarid1b O/E *versus* control ^#A^*P*<0.01 ^#B^*P*<0.05, Jarid1b O/E *versus* Jarid1b+Ship1 O/E **P<0.01, *P<0.05, Student's *t*-test. (**e**) Hypothetic model explaining how JARID1B may contribute to HPSCC differentiation

Theoretically, Jarid1b upregulation leads to transcription inhibition of its target genes. Thus, we speculated that the target genes of Jarid1b should be any inhibitors of PI3K-AKT pathway and Pten or Ship1 could be potential candidates. We first examined Pten and Ship1 mRNA expression levels by RT-qPCR. The results demonstrated that at transcription level Ship1 was substantially downregulated by Jarid1b overexpression whereas Pten only slightly decreased (Figure 5a). Chromatin immunoprecipitation (ChIP) assay was performed to validate whether Jarid1b controls *Ship1* transcription by directly binding *SHIP1* gene promoter. We designed five pairs of primer targeting the promoter and intron 1 of *SHIP1* gene as indicated in Figure 5b. The results demonstrated that Flag-Jarid1b was enriched at transcription start site (TSS) and promoter region of *SHIP1* gene (Figure 5b). H3K4me3 enrichment also showed a similar pattern in the Jarid1b O/E cells (Supplementary Figure S5A). Moreover, H3K4me3 enrichment was reduced at *SHIP1* gene TSS upon Jarid1b overexpression (Figure 5b). The results indicate that Jarid1b controlling Ship1 expression could be associated with its demethylase function.

### Rescue of Jarid1b-overexpressing phenotypes by Ship1 in FaDu cells

We next asked that if overexpression of Ship1 could rescue Jarid1b-induced phenotypes. The results showed that overexpression of Ship1 could attenuate the elevation of K10 expression induced by Jarid1b (Figure 5c). Furthermore, the inhibition of cell growth induced by Jarid1b got restored by the overexpression of Ship1 (Figure 5d). Together, the results suggest that Ship1 is the direct target of Jarid1b to induce FaDu cell differentiation by activating Ship1-PI3K-Akt pathway.

## Discussion

Although Jarid1b overexpression occurs in a wide variety of cancers, the function of Jarid1b in cancer is not fully understood. Epigenetic mechanisms have been documented as a critical step in tumorigenesis, progression and metastasis, but how these epigenetic molecules exactly control the downstream pathway or whether the phenomenon simply occurs concomitantly is still underexplored. Here, for the first time, we uncovered the relevance of Jarid1b, a demethylase of H3K4me3, in control of squamous cancer cell commitment. We showed that elevated Jarid1b promotes the HPSCC differentiation and inhibits cancer cell proliferation. Importantly, we dissected the molecular mechanisms of this regulation by showing that Jarid1b could induce K10 expression by controlling its downstream target gene, Ship1, an inhibitor of PI3K-AKT pathway (Figure 5e).

Epigenetic modification has a critical role in the maintenance of cell fate.^29^ ESCs, progenitors and cancer stem cells are characterized by distinct epigenetic features to maintain the differentiation potential. Among these are an activate histone mark, H3K4me3, and a repressive mark H3K27me3, which are largely enriched at the promoter and mark developmental and lineage-specific genes.^30^ Our previous results have showed that removal of Ezh1 and Ezh2, key Polycomb subunits, from mouse skin leads to remarkable switch in fate determination in epidermal progenitor cells, resulting in an increase in the number of lineage-committed Merkel cells.^31^ The role of the Jarid1b in controlling cancer cell commitment is somehow reminiscent of its function in breast cancer cells, where Jarid1b has also been shown to drive a luminal transcriptional program.^22^ Here we provided first evidence that overexpressed Jarid1b induces cancer differentiation in HPSCC.

It has been reported that Jarid1b functions as an oncogene in a variety of tumors. The proliferation of cancer cells is inhibited when depleting Jarid1b. Interestingly, we observed that overexpression of Jarid1b in FaDu cells could inhibit the proliferation, which suggests that the function of Jarid1b is more complicated than we thought. Here we mainly ectopically expressed Jarid1b to investigate the role of Jarid1b, whereas most of the previous results were obtained by the depletion of Jarid1b. Epigenetic regulators are ubiquitously expressed and have a fundamental role in the cells and are involved in cell cycle dynamics. In hepatocellular carcinoma, gastric cancer and lung cancer Jarid1a suppresses the expression of CDK inhibitors p21Cip1, P27Kip1 and P16Ink4a.^32, 33, 34, 35^ In addition, Jarid1b can repress the cell cycle inhibitor p21Cip1 in a variety of cell lines.^36^ In our study we found that the FaDu cell proliferation can also be inhibited upon the overexpression of Jarid1b. By dissecting the genetic mechanism, we demonstrated that Jarid1b promotes differentiation and inhibits proliferation of FaDu cells by repressing Ship1, a phosphatase of PIP3. Ectopic expression of Ship1 results in the de-differentiation and improves the proliferation of differentiated FaDu cells, indicating that Ship1 is a critical regulator of FaDu cell differentiation. Our results showed that Jarid1b directly inactivates Ship1, one of critical regulator of PI3K/AKT pathway. Concordant inhibition of PI3K/AKT attenuates the Jarid1b overexpression induced phenotypes, confirming the importance of Jarid1b repression of Ship1 in promoting FaDu cell differentiation state. Recently, other investigations have implied that other members of Jarid1 family serve as tumor suppressor. Jarid1c is a target gene of von Hippel Lindau (VHL) tumor suppressor protein. Depletion of Jarid1c accelerates tumor growth in VHL-deficient cells.^37^ Jarid1a has been described as a binding partner of the retinoblastomatumor suppressor protein. Jarid1a and Jarid1b contribute to a tumor suppressor network controlling cellular senescence.^38^ Jarid1d, a male-specific protein, represses invasion-associated genes MMP1, MMP2, MMP3, MMP7 and Slug *in vitro* and *in vivo*. Low JARID1D level is associated with poor prognosis in prostate cancer patients.^39^ We still do not know whether this function of Jarid1b could apply to other cancers or it is heavily context-dependent. However, this study sheds light on the importance of Jarid1b as a tumor suppressor at least in squamous cancers.

Hyperactivation of the PI3K/Akt pathway is a universal tumor driver which correlated with tumor cellular functions, such as proliferation, metabolism, invasion, metastasis and survival. Thus, PI3K signaling could be an important therapeutic target and several small molecules have been developed to antagonize activity of the pathway. However, some results from PI3K/AKT inhibitor trials suggest that deep and sustained responses to single agents are infrequent. Moreover, Caino *et al.*^40^ have reported that PI3K inhibitors currently in clinic induce global transcriptional reprogramming in tumors which improve the cell invasion and motility. Therefore, a deeper understanding of function of PI3K/AKT pathway in diverse tumors needs to be uncovered. Interestingly, recent report has shown that all-trans retinoic acid modulates osteogenic differentiation of mesenchymal stem cells via activating the PI3K/AKT/*β*-catenin signaling pathway.^41^ Here we described the Jarid1b-Ship1-PI3K network, which is associated with cancer differentiation grade. The function of Jarid1b-regulatory network needs to be explored in other types of tumor in the future studies.

In summary, we described the novel molecular function of Jarid1b in HPSCC. Our data support a key role for Jarid1b in improving cell differentiation in HPSCC, where it has been implicated to function as a tumor suppressor inhibiting cell proliferation in a Ship1-dependent manner. These new insights into the role of Jarid1b in tumor may develop potential treatment strategies for the HPSCC.

## Materials and Methods

### Cell culture and treatment of inhibitors

FaDu cells were obtained from ATCC and cultured in DMEM medium with 10% fetal bovine serum and 1% penicillin/streptomycin. Cells were treated with inhibitors (Selleck, Shanghai, China) at indicated concentration for 24 h.

### Plasmids

Flag-Jarid1b was subcloned into PiggyBac vector and transfected with help vector. To get the stable and pure Jarid1b-overexpressing cell population we performed selection with 300 *μ*g/ml of hygromycin until the control cells totally died. Ship1 was subcloned into pcDNA3-Flag vector. For the immunofluorescence staining we subcloned Jarid1b-Flag to the pLenti-GIII-CMV-IRES-puro-SV40-GFP vector. To get pure cell population 2 *μ*g/ml puromycin selection was performed after 24 h of transfection. Then we changed to new medium after 12 h when the blank control cells died totally. Visually all the cells were GFP positive under fluorescence microscope monitor. Jarid1b shRNA plasmids were obtained from JiKai Company. The target sequences could be found in the Supplementary Table. The plasmids were transfected with Lipofectamine 3000 into cells following the instructions from the manufacturer.

### Western blot analysis

Total cellular or tissue lysates were prepared with 1% SDS and sonicated to break up DNA. Antibody data can be found in the Supplementary Table. Each immunoblot was repeated three times containing samples coming from different cell cultures. The relative intensity of protein bands was measured with NIH image J software. The blots were developed with Super Signal Pico substrate (Pierce Biotechnology, Shanghai, China).

### Real-time reverse-transcription PCR

Total RNA was extracted from cells or tissues using RNAiso Plus (Takara D9108, Dalian, Liaoning, China) following the instructions from the manufacturer. RNA concentration was determined by Nanodrop. Reverse procedure of cDNA was done using First-strand cDNA Synthesis kit (Takara RR037A). RT-qPCR was performed using the Roche Lightcycler480 Real-time PCR System with SYBR green reagents from Takara (RR820A). Genes were amplified using the primers described in Supplementary Table. Quantifications were normalized to GAPDH for cell or 18 s rRNA for tissue.

### ChIP

ChIP assay was performed as described.^24, 31^ In brief, cells were cross-linked using 1% formaldehyde for 10 min at room temperature and then rinsed twice with 1 × PBS. Cells were lysed and sonicated to shear cross-linked DNAs. The sheared DNA samples were incubated for at least 16 h with 100 *μ*l of Dynal Protein G magnetic beads (Thermo 1003D, Shanghai, China), which has been preincubated with antibodies overnight at 4 °C. After ChIP, each sample was washed once in turn with 1 ml low-salt, high-salt, LiCl and TE buffer for 45 min at 4 °C. Bound complexes were eluted and reverse cross-linked by overnight incubation at 65 °C in the presence of RNase A and proteinase K. Finally, the DNA was purified with Phenol/chloroform/isoamyl alcohol as described.^42^

### Immunohistochemistry

Citrate buffer, pH 6.0, was used to unmask the epitopes of paraffin-embedded samples. Primary antibodies against Jarid1b and K10 were incubated overnight at 4 °C. Mean Intensity and the percentage (0–100%) were quantified by Image-Pro Plus from at least three raw, single-channel gray scale images, which were conducted at the same condition.

### Immunofluorescence

Cells were fixed for 10 min in 4% PFA and blocked for 1 h with blocking buffer. Primary antibody was diluted in blocking solution and incubated overnight at 4 °C. After washing, secondary antibodies were incubated for 1 h at room temperature. Then DAPI was counterstained for 5 min. Finally the cells were mounted using anti fade mounting media.

### *In vitro* proliferation assay

*In vitro* cell proliferation assay was carried out using a Cell Counting Kit-8 (Dojindo CK04, Shanghai, China) following the manufacturer's instructions. In brief, the cells were incubated for 30 min to 2 h after adding 10 *μ*l CCK8 solutions in 100 *μ*l medium. Then measure the absorbance at 450 nm using a microplate reader.

### Statistical analysis

To determine the significance of data obtained from human samples or cell culture assays, comparisons were made using descriptive and inferential statistics accompanied by graphs from Prism software program (GraphPad, La Jolla, CA, USA). Western blot analyses were normalized to *β*-tublin protein. In all column bar graphs, mean value±1 s.d. is presented. For all the statistics the 0.05 level of confidence was accepted for statistical significance.

## Abbreviations

HPSCC: hypopharyngeal squamous cell carcinoma
ChIP: chromatin immunoprecipitation
IHC: immunohistochemistry
Tan IIA: tanshinone IIA
O/E: overexpressing
