# Supplementary figures and images for: Overexpression of JARID1B promotes differentiation via SHIP1/AKT signaling in human hypopharyngeal squamous cell carcinoma
Source: Cell Death Dis. 2016 Sep 1;7(9):e2358–. doi: 10.1038/cddis.2016.262 (PMC5059865; doi:10.1038/cddis.2016.262)

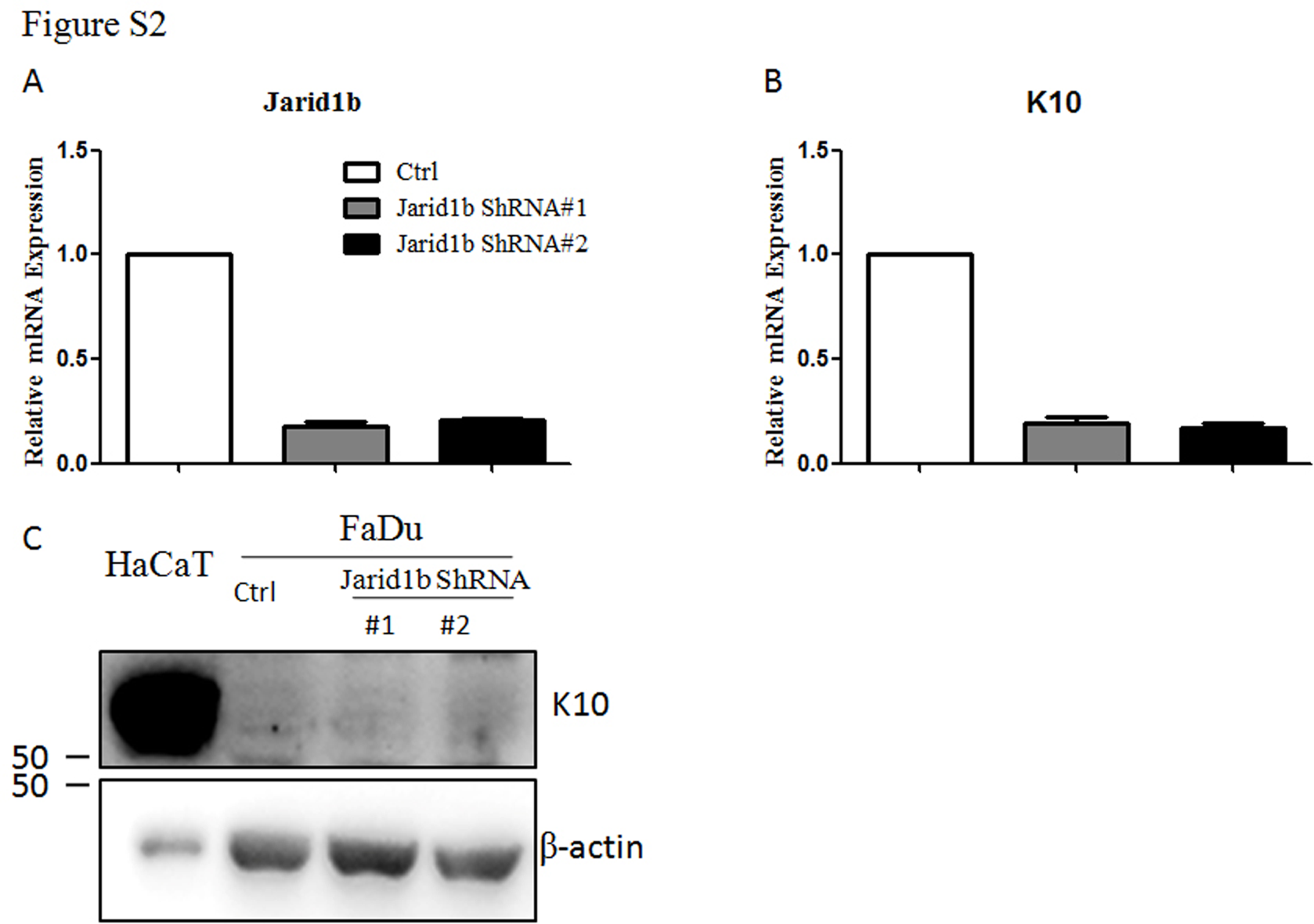

Supplement: Supplementary Figures S3 [file cddis2016262x1.tif]

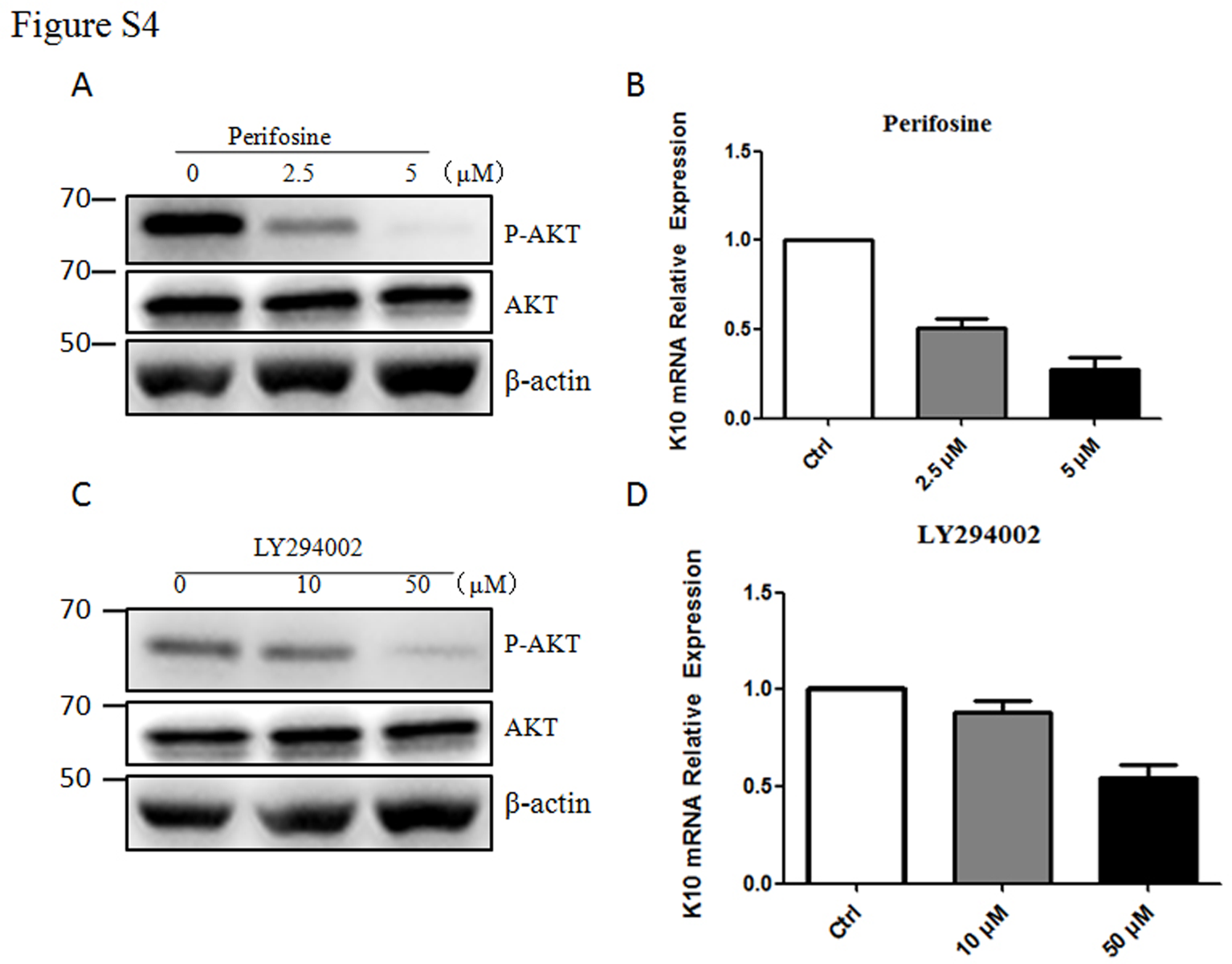

Supplement: Supplementary Figures S4 [file cddis2016262x2.tif]

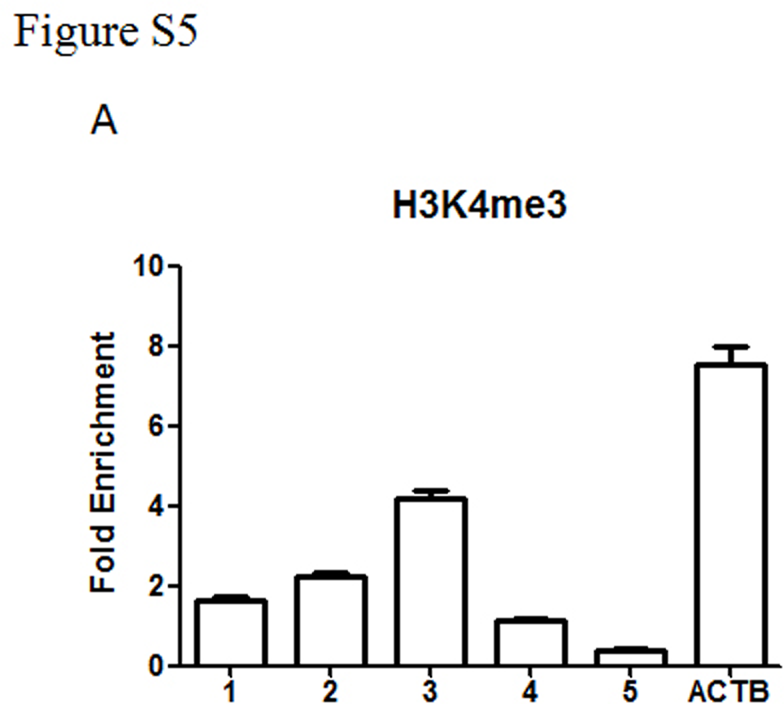

Supplement: Supplementary Figures S5 [file cddis2016262x3.tif]
